# Genetic Diversity and Distribution of* Blastocystis* Subtype 3 in Human Populations, with Special Reference to a Rural Population in Central Mexico

**DOI:** 10.1155/2018/3916263

**Published:** 2018-03-18

**Authors:** Liliana Rojas-Velázquez, Patricia Morán, Angélica Serrano-Vázquez, Leonardo D. Fernández, Horacio Pérez-Juárez, Augusto C. Poot-Hernández, Tobías Portillo, Enrique González, Eric Hernández, Oswaldo Partida-Rodríguez, Miriam E. Nieves-Ramírez, Ulises Magaña, Javier Torres, Luis E. Eguiarte, Daniel Piñero, Cecilia Ximénez

**Affiliations:** ^1^Unidad de Investigación en Medicina Experimental, Facultad de Medicina, Universidad Nacional Autónoma de México (UNAM), Dr. Balmis 148, Doctores, Cuauhtémoc, 06726 Ciudad de México, Mexico; ^2^Unidad de Posgrado, Universidad Nacional Autónoma de México (UNAM), Circuito de Posgrado S/N, Coyoacán, Cd. Universitaria, 04510 Ciudad de México, Mexico; ^3^Centro de Investigación en Recursos Naturales y Sustentabilidad (CIRENYS), Universidad Bernardo O'Higgins, Avenida Viel 1497, Santiago, Chile; ^4^Departamento de Ingeniería de Sistemas Computacionales y Automatización, Sección de Ingeniería de Sistemas Computacionales, Instituto de Investigaciones en Matemáticas Aplicadas y en Sistemas, Universidad Nacional Autónoma de México (UNAM), Circuito Escolar 3000, Cd. Universitaria, Coyoacán, 04510 Ciudad de México, Mexico; ^5^Unidad de Bioinformática, Bioestadística y Biología Computacional, Red de Apoyo a la Investigación, Coordinación de la Investigación Científica, UNAM, Instituto Nacional de Ciencias Médicas y Nutrición, Vasco de Quiroga 15, Tlalpan, 14080 Ciudad de México, Mexico; ^6^Unidad de Investigación Médica en Enfermedades Infecciosas y Parasitarias, Hospital de Pediatría, Centro Médico Siglo XXI, Instituto Mexicano del Seguro Social (IMSS), Avenida Cuauhtémoc 330, Doctores, Cuauhtémoc, 06720 Ciudad de México, Mexico; ^7^Departamento de Ecología Evolutiva, Instituto de Ecología, Universidad Nacional Autónoma de México (UNAM), Circuito Exterior S/N, Junto al Jardín Botánico, Cd. Universitaria, Coyoacán, 04510 Ciudad de México, Mexico

## Abstract

*Blastocystis* subtype 3 (ST3) is a parasitic protist found in the digestive tract of symptomatic and asymptomatic humans around the world. While this parasite exhibits a high prevalence in the human population, its true geographic distribution and global genetic diversity are still unknown. This gap in knowledge limits the understanding of the spread mechanisms, epidemiology, and impact that this parasite has on human populations. Herein, we provided new data on the geographical distribution and genetic diversity of* Blastocystis* ST3 from a rural human population in Mexico. To do so, we collected and targeted the SSU-rDNA region in fecal samples from this population and further compared its genetic diversity and structure with that previously observed in populations of* Blastocystis* ST3 from other regions of the planet. Our analyses reveled that diversity of* Blastocystis *ST3 showed a high haplotype diversity and genetic structure to the world level; however, they were low in the Morelos population. The haplotype network revealed a common widespread haplotype from which the others were generated recently. Finally, our results suggested a recent expansion of the diversity of* Blastocystis* ST3 worldwide.

## 1. Introduction

*Blastocystis *(Heterokonta, Stramenopiles) is a genus comprising parasitic protists that inhabit the digestive tract of several metazoans, such as fishes, amphibians, birds, reptiles, rodents, and humans [1–3].* Blastocystis *is globally distributed, showing a high rate of infection from underdeveloped to developed countries [4, 5].

This parasite is often transmitted via the oral-fecal route to people who work directly with animals, such as those involved in intensive animal farming or industrial livestock production [6]. In humans, the signs and symptoms associated with* Blastocystis* infection range from diarrhea to flatulence, bloating, and abdominal discomfort [7, 8], with the “irritable bowel syndrome” (IBS) being the most frequent clinical manifestation [8–11].

Molecular evidence based on the small subunit ribosomal RNA (SSU-rDNA) gene suggests that, at least, 17 genetic subtypes can be recognized within* Blastocystis* [12]. Nine of these subtypes are found in humans, with subtype 3 (ST3 hereafter) being the most common in epidemiological studies worldwide [12–18]. ST3 has been regarded to trigger IBS in humans [8], and recent research also suggests an association between this subtype and colorectal cancer [19]. Other studies, however, suggest a lack of association between the ST3 and some type of symptomatology in humans [7, 20]. While* Blastocystis* ST3 has medical importance and high prevalence in humans, the real magnitude of its genetic diversity and geographical distribution remains so far unknown [5, 17]. Apparently, the ST3 exhibits a broader geographical distribution and higher genetic diversity than other genetic subtypes of* Blastocystis* [16, 21], but this hypothesis still needs to be tested using genetic data and clinical cases from both well-studied and undersampled geographical areas. In this context, there are very few geographical and genetic data on* Blastocystis* ST3 from Mexico.

Herein, we aimed to provide new data on the geographical distribution and genetic diversity of* Blastocystis* ST3 from a rural and asymptomatic human population in Mexico. To do so, we collected and targeted the SSU-rDNA region in fecal samples from this population and further compared its genetic diversity and structure with those previously observed in populations of* Blastocystis* ST3 from other regions of the planet.

## 2. Materials and Methods

### 2.1. Ethical Considerations

The protocol used in this study was conducted under the ethical principles and approval of both the Mexican Commission on Ethics and Research of the Health Ministry of the State of Morelos (Comisiones de Ética y de Investigación del Ministerio de Salud del Estado de Morelos) and the Commission on Ethics in Research of the Facultad de Medicina of the Universidad Nacional Autónoma de México (UNAM) (Comité de Ética de Investigación de la Facultad de Medicina de la Universidad Nacional Autónoma de México). The guidelines of the committees are based on the Mexican Official Norm (Norma Oficial Mexicana NOM-012-SSA3-2007), which regulates the ethical principles of every research on humans and on laboratory animals, as well as on the Declaration of Helsinki, which set ethical principles regarding human experimentation developed by the World Health Organization (WHO).

Based on the abovementioned guidelines, our study only used samples from volunteers, who were respectively informed about the objectives of this research, the potential risks (if any), and the sampling procedures. We obtained an informed consent letter from all the participants.

### 2.2. Sampling and Analysis

Between May and November 2015, fecal samples were collected from 182 volunteers (86 males and 96 females) from Puente de Ixtla in the community of Xoxocotla, State of Morelos (Mexico), ranging in age from 2 to 51 years old. The asymptomatic status was defined according to the ROME III criteria. Three fecal samples were collected from each volunteer on three consecutive days. The samples were maintained at 4°C and transported to the laboratory in Mexico City on the same day of collection. A subsample of each fecal sample was smeared, stained with 4% Lugol's iodine solution, and examined under a light microscope at 10x and 40x magnifications [22].

### 2.3. Amplification and Sequencing of SSU-rDNA

DNA was extracted from fresh fecal samples using QIAamp DNA stool kit (QIAGEN, Hilden, Germany) and following the manufacturer's instructions. PCR protocol targeting the SSU-rDNA was conducted according to Scicluna et al. [23]. In brief, we used a total mixture of 20 *μ*l : 20 *μ*M of primers RD5 (5′-ATC TGG TTG ATC CTG CCAG T-3′) and BhRDr (5′-GAG CTT TTT AAC TGC AAC AAC G-3′) [23], as well as 0.025 U of polymerase (AmpliTaq Platinum Polymerase, Invitrogen). To verify the presence of a single band and the size of the amplified products (approximately 600 bp), the PCR products were separated by electrophoresis in agarose gel (1.5%) in the presence of ethidium bromide, visualized by ultraviolet transillumination, and photographed. The amplification product of a 600 bp fragment of the* Blastocystis* SSU-rDNA was purified and sequenced using a dideoxynucleotide-terminal method. Sequencing was carried out in a capillary sequencer (ABI-Avant 100, University of Washington). The sequences obtained were edited and/or analyzed with BioEdit, MEGA 5.0 software [24, 25], and ad hoc scripts from Python. These sequences were compared to sequences available in GenBank, employing BLAST to establish their identity. The final sequences were deposited in GenBank under accession numbers MF539962–MF540015.

### 2.4. Global Genetic Diversity and Haplotype Network for* Blastocystis* ST3

We investigated the global genetic diversity (i.e., Latin America, Europe, and Asia) within* Blastocystis* ST3 using the novel SSU-rDNA sequences reported in the present study and those previously reported within the literature. We provided an exhaustive list of the latter sequences (*n* = 169) and sources in the Supplementary Material (available here). We investigated the following descriptive statistics of genetic diversity using the software DnaSP ver. 5.10.01 [26]: number of segregating sites (*S*), number of haplotypes (*h*), haplotype diversity (Hd), and nucleotide diversity (*π*) for each set of sequences according to their geographical region of origin. We built a global haplotype network using TCS network inference method [27] implemented in the PopART program ver. 1.7 (http://popart.otago.ac.nz/downloads.shtml) to investigate the global genealogical relationship between the different haplotypes of* Blastocystis *ST3. We also ran Tajima's *D* test in the software DnaSP [26] to investigate possible events of global population expansion on* Blastocystis *ST3. We finally estimated pairwise *F*_ST_ statistics in the software Arlequin ver. 3.11 (http://cmpg.unibe.ch/software/arlequin3) to investigate whether the geographical populations of* Blastocystis *ST3 were genetically structured.

## 3. Results

### 3.1. Frequency of* Blastocystis* ST3 in Morelos, Mexico

**Figure 1:**
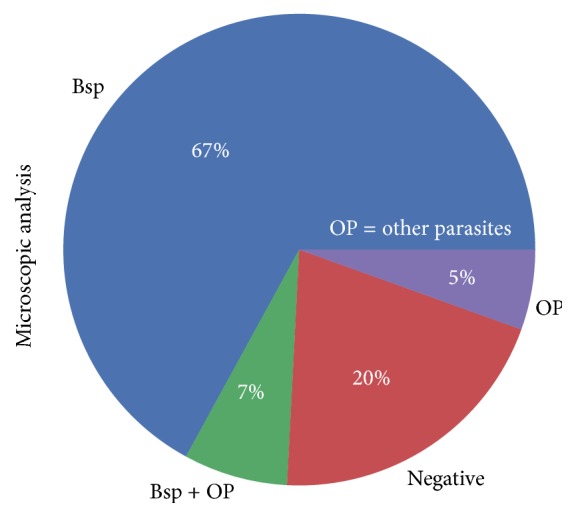
Frequency of intestinal parasites based on microscopic analysis of the fecal samples taken in Morelos, Mexico.* Blastocystis* was the only parasitic infection found in 67% of individuals and in 7% in coinfection with other parasites. Bsp:* Blastocystis*; OP: parasites other than* Blastocystis*; BSP + OP: coinfection of* Blastocystis* and other parasites; Negative: no parasite found. Among OP: Chm,* Chilomastix mesnili*; Ec,* Entamoeba coli*; En,* Endolimax nana*; Hn,* Hymenolepis nana*; Gl,* Giardia lamblia*; Ib,* Iodamoeba bütschlii*.

A microscopic analysis revealed that 148 (81.32%) of the 182 fecal samples collected in Morelos (Mexico) exhibited at least some type of intestinal parasite. These 148 samples (positive samples hereafter) harbored different parasites, including representatives of* Blastocystis, Chilomastix mesnili, Entamoeba coli, Hymenolepis nana, Iodamoeba bütschlii, Endolimax nana, *the* Entamoeba histolytica/Entamoeba dispar* complex, and* Giardia lamblia*. Among the abovementioned parasites,* Blastocystis* had the greatest frequency, occurring in 109 (74%) out of 148 positive samples. It was also the unique parasite in 99 (67%) out of 148 positive samples, and 7% of the samples (10/148) were coinfected with other parasites (Figure 1).

**Figure 2:**
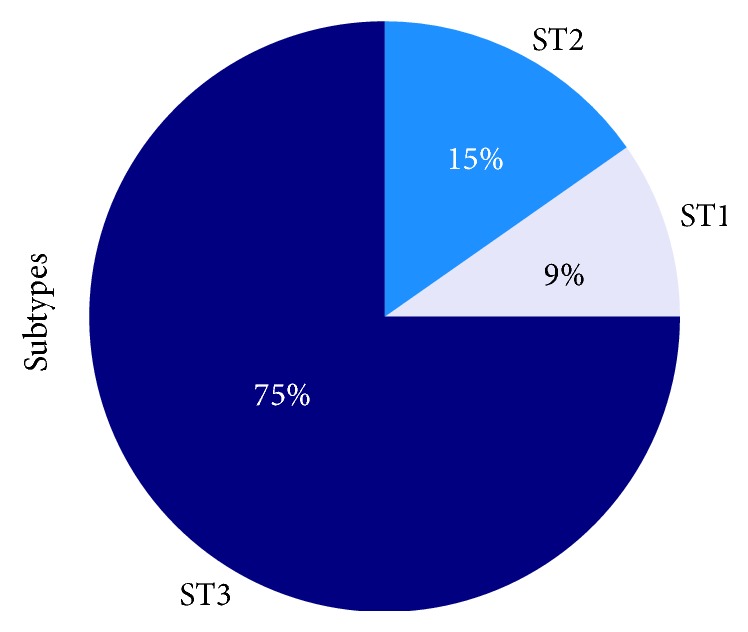
Frequency of* Blastocystis* subtypes in the study population. Targeting the SSU-rDNA according to DNA-barcoding, Three* Blastocystis* subtypes (ST) were recorded according to the following frequencies:* Blastocystis* ST1, 9.7% (*n* = 7 samples); ST2, 15.3% (*n* = 11 samples); and ST3, 75% (*n* = 54 samples).

Further PCR and sequencing procedures successfully confirmed the presence of three different* Blastocystis* subtypes in 72 of the 148 positive samples collected in Morelos. These three* Blastocystis* subtypes (ST) were recorded according to the following frequencies:* Blastocystis* ST1, 9.7% (*n* = 7 samples); ST2, 15.3% (*n* = 11 samples); and ST3, 75% (*n* = 54 samples) (Figure 2).

### 3.2. Genetic Diversity of ST3 and Haplotype Network

Genetic diversity indices revealed a total of 44 segregating sites (*S*) and 20 haplotypes (*h*), as well as a total haplotype diversity (Hd) of 0.563 and nucleotide diversity (*π*) of 0.019. Tajima's *D* test provided values ranging between −1.303 and −2.363 (Table 1). A pairwise *F*st analysis revealed that there is very low genetic differentiation between all geographical populations of* Blastocystis *ST3 (Table 2).

The number of haplotypes ranged from 3 to 15 between human populations, the number of segregating sites ranged between 1 and 35, haplotype diversity ranged between 0.142 and 0.740, and nucleotide diversity ranged between 0.001 and 0.045 (Table 1). The ST3 genetic diversity of Latin American populations (except Morelos's population) and Eurasia exhibited the highest values of genetic diversity indices in contrast to Morelos's population, where low haplotype diversity (three haplotypes) was detected (Table 1).

**Figure 3:**
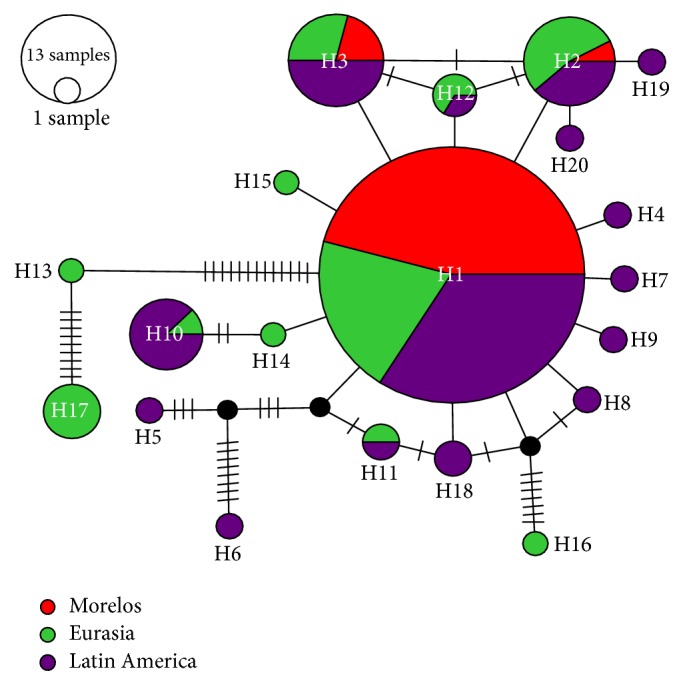
Haplotype network of* Blastocystis* ST3 of human populations at different regions from Latin America, Europe, and Asia. Each circle represents a haplotype, and each color represents the place where it was obtained. The size of each circle is proportional to the frequency of the haplotype in each population, where it was found. The circles in black stand for missing haplotypes and the short lines show the mutational steps.

The haplotype network showed the haplotype distribution of the ST3 (Figure 3). In general, the worldwide haplotype network evidenced large levels of diversity, with a total of 20 haplotypes, and haplotype 1 was the dominant. The network showed a star topology radial distribution (Figure 3). Also, haplotype 1 was the most frequently found in Morelos's population and this haplotype is commonly distributed in American populations.

## 4. Discussion

In the present study, we analyzed the frequency and distribution of* Blastocystis* subtypes in an asymptomatic rural population. The results revealed a great frequency of* Blastocystis* from 74%, above the national average, as the frequency of this parasite varies from 23% to 61% in Mexico [20, 28, 29]. Recent studies in South America have described similar frequency of* Blastocystis* in the human population (21% to 67%) [30–32]. Around the world,* Blastocystis* exhibits a frequency range of 0.5% to 62% [4]. The higher prevalence of* Blastocystis* has been linked to hygiene factors including the consumption of food/water contaminated with* Blastocystis* and exposure to domestic and peridomestic animals infected too with this parasite [4, 33].

Many years ago,* Blastocystis *was considered as saprophytic yeast of the digestive tract, innocuous for the host [34]. Nowadays, we can observe that this parasite is widely distributed in the human population in the world and it is similarly distributed in symptomatic and asymptomatic individuals. For instance, Morelos's population (Mexico) showed a high infection frequency of* Blastocystis* although the participants were asymptomatic, suggesting tolerance to this parasite, as reported elsewhere [20, 35, 36].

*Blastocystis *has a high worldwide genetic diversity, represented by 17 subtypes (ST1–ST17) [5]. It is possible that there are other subtypes capable of infecting humans and other vertebrates [4, 5]. Regarding the distribution of subtypes in the present study, ST1 (9.7%), ST2 (15.3%), and ST3 (75%) were identified among 72 human isolates successfully genotyped. Globally,* Blastocystis* ST3 is the most prevalent subtype in humans found in different geographic areas [5, 37]. In our study, the frequency of ST3 was 75%, high compared to other populations of Mexico as the state of Michoacan, where the frequency of this subtype was 21%, and in Mexico City it was 42% [38, 39].

In Latin America, the frequency of ST3 is high [4, 37], with the following frequencies reported for* Blastocystis *ST3 in this political region: 14% in Colombia, 36% in Brazil, 84% in Ecuador, 30% in Bolivia, 92% in Peru, and 63% in Argentina [40]. While ST3 is amply distributed worldwide, it is more prevalent in Latin America [37], which opens the possibility that this subtype was generated in this geographic area and spread to the rest of the continents.

To improve our knowledge on the magnitude of the genetic diversity of* Blastocystis* ST3 on the planet, we analyzed sequences of Mexico City, South America, France, Switzerland, Italy, Nepal, and Iraq and the sequences obtained in the present study (169 total sequences, 54 from Morelos and 115 from the NCBI database). The genetic parameters calculated for these sequences suggested a recent population increase or directional/purifying selection. These results are supported by the haplotype network, which showed a star topology with the haplotypes distributed in a radial way supporting inferences of a recent geographical expansion in* Blastocystis* ST3. This behavior is similar to reporting by other parasites, such as* Plasmodium falciparum* [41].

A total of 20 haplotypes were found on all sequences analyzed. Haplotype 1 was the most abundant and widely distributed, being detected in the majority of the studied countries, mainly in Latin America. These results are showing that haplotype 1 is perhaps an ancestral type from which all the other haplotypes have been generated recently. Haplotype 1 probably originated in Latin America and has recently colonized other regions of the world, probably via human migration [42].

In some studies, it has been observed that this subtype has been strongly related to rural populations [8, 43]. It is possible to hypothesize that the migration phenomenon [42] occurs mainly from the displacement of these rural communities infected with* Blastocystis* ST3 haplotype 1 spreading this parasite into the cities. Already in large cities, the transmission of* Blastocystis* ST3 haplotype 1 could happen from one person to another [43]. In addition, due to the opening of political borders between developed countries, this process of travel-associated infection is common, because travelers may act as carriers of this parasite [44].

Also, it is possible that the other haplotypes of* Blastocystis* ST3 could be colonizing different areas promoted by the human migratory phenomenon. This idea would explain the high representation of certain haplotypes throughout the American continent and others in European countries. For example, seven sequences of haplotype 9 correspond to South America and only one corresponds to Europe (Switzerland).

However, to test both hypotheses, it will be necessary to have a larger number of sequences and complete genomes from around the world. It is known that migration causes mobility of parasites and it is important to have information of other sociodemographic parameters of the host as the nationality or antecedents of previous travels to endemic areas [45]. In addition, the distribution of* Blastocystis* could be related to the lack of symptoms that occurs in many cases, because asymptomatic* Blastocystis* infections are not treated and therefore there are a large number of carriers of this parasite. It is known that these factors can influence the distribution of parasites [42, 46].

With respect to Morelos, all the sequences of* Blastocystis* ST3 were grouped into three haplotypes, which means that there is a low genetic diversity, a reduced rate of mutations, and little genetic differentiation; this suggests isolation and homogeneity in the population.

## 5. Conclusions

To our knowledge, this is the first study analyzing the haplotype diversity and distribution of* Blastocystis* ST3 subtypes in different human populations. In addition, our work facilitates the vision of a global distribution in* Blastocystis* ST3. We provide evidence of a recent expansion of this subtype that may be related to the migration of humans to other regions of the world. However, it is necessary to continue studying this parasite, in order to generate a more complete knowledge that allows us to know the course of* Blastocystis* infection, its epidemiology, and the causal factors that contribute to its dispersion dynamics and distribution.

## Figures and Tables

**Table 1:** Statistics data of genetic diversity observed within different geographical populations of *Blastocystis* ST3 around the world.

Populations	*N*	*S*	*h*	Hd	*π*	±SD	Tajima's *D*
Morelos	54	1	3	0.14186	0.00107	0.068	−1.68258^ns^
Latin America	69	25	15	0.67775	0.01334	0.058	−2.36261^*∗∗*^
Eurasia	46	35	11	0.74010	0.04473	0.052	−1.30307^ns^
All populations	169	44	20	0.56276	0.01886	0.044	−2.20000^*∗∗*^

*N*: number of sequences; *S*: number of segregating sites; *h*: number of haplotypes; Hd: haplotype diversity; *π*: nucleotide diversity; ns: not significant. ^*∗∗*^*p* < 0.01. Latin America: *Blastocystis* populations of North and South America (i.e., Mexico, Colombia, Brazil, Ecuador, Bolivia, Peru, and Argentina), except that of Morelos. Eurasia: *Blastocystis* populations of Europa and Asia (i.e., Nepal, Switzerland, Iraq, Italy, and France).

**Table 2:** Estimates of *F*_ST_ based on the SSU-rDNA variation observed between different geographical populations of the parasite *Blastocystis* ST3.

Population	Morelos	Latin America	Eurasia
Morelos	------		
Latin America	0.04165^ns^	------	
Eurasia	0.09164^ns^	0.05975^ns^	------

Latin America: *Blastocystis* populations of North and South America (i.e., Mexico, Colombia, Brazil, Ecuador, Bolivia, Peru, and Argentina), except that of Morelos. Eurasia: *Blastocystis* populations of Europe and Asia (i.e., Nepal, Switzerland, Iraq, Italy, and France). Probability obtained by a permutation test with 50,000 replicates. ns: not significant.

## Supplementary Materials

Supplementary Materials List of the sequences reported in the present study and those previously reported within the literature.
